# 25, 50 & 75 years ago

**DOI:** 10.1111/ans.18127

**Published:** 2022-11-17

**Authors:** Julian A. Smith

**Affiliations:** ^1^ Department of Surgery Monash University Melbourne Victoria Australia

## Twenty‐five years ago

**Clarnette TD, Beasley SW. Handlebar injuries in children: patterns and prevention. *ANZ J. Surg*. 1997; 67: 338–9.**

**Fig. 1:**
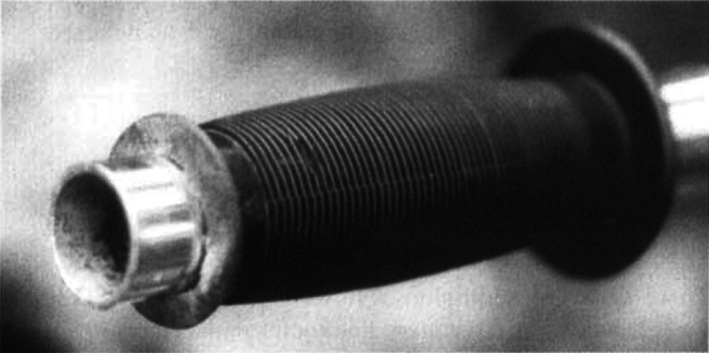
The sharp chromic end of the handlebar has cut through the softer rubber handle‐grip which then tends to migrate medially.

Bicycle handlebar injuries in children are a significant cause of abdominal trauma. The present study documents 32 children with handlebar injuries who were managed at the Royal Children's Hospital over a 5‐year period, and suggests a design change to bicycle handlebars which may reduce the severity of injury. A retrospective review of all the children admitted to the Royal Children's Hospital with handlebar injuries between January 1990 and January 1995 was undertaken. The age, sex, nature of injury, length of hospital stay and management were recorded. Thirty‐two children with blunt abdominal trauma or lacerations resulting from handlebar injuries were identified. Injuries included: splenic trauma (9); liver trauma (4); traumatic pancreatitis (3); transection of the pancreas (2); renal contusions (2); duodenal haematoma (l); and bowel perforation (3). In addition, there were three urethral injuries and five lacerations involving the abdominal wall and inguinoscrotal region. The presence of external bruising was a poor indicator of underlying organ damage. Thirteen operations were performed and the mean hospital stay for the series was 9 days. Handlebar injuries are a significant cause of both blunt abdominal trauma and lacerations to the contact area. The infrequent finding of external bruising in the presence of major organ damage suggests that, although the velocity at impact may be relatively low, the small cross‐sectional area of the end of the handlebar is a major factor contributing to organ damage. Moreover, we suspect that the high proportion of lacerations observed in this type of trauma results from the sharp metallic end of the handlebar cutting through the soft rubber handle (Fig. 1). Manufacturers of bicycles should be made aware of these findings and should adjust the design of the handlebars accordingly.

**Pillay SP, Wynter C, Lynch S, Wall D, Balderson G, Strong R. Endotoxin levels in donors and recipients during orthotopic liver transplantation. *ANZ J. Surg*. 1997; 67: 187–91**.

The hypothesis being tested in this paper is that endotoxin levels in donors and in recipients during liver transplantation influences postoperative outcome. Endotoxin levels in systemic venous and portal venous blood were measured in 46 adult donors and 44 adult recipients (47 liver transplants) during the period 1992–1995. Endotoxin was measured using a modification of the *Limulus* amoebocyte lysate (LAL) assay. In the donor, systemic endotoxin levels were above normal levels at 10.0 ± 1.3 pg/mL from the start and rose to 15.8 ± 2.9 pg/mL after dissection of the hilar structures, but fell to 10.6 ± 0.8 pg/mL just prior to the removal of the liver (control = 7.8 pg/mL). The mean portal venous endotoxin levels were 18.2 ± 3.4 pg/mL after dissection of the hilar structures and I 2.6 ± 0.9 pg/mL after cannulation of the portal vein. In the recipients, the highest level in the portal venous blood occurred at the end of the anhepatic phase (46.5 ± 6.7 pg/mL). The systemic venous samples in the recipients were elevated to start with, but fell rapidly to 19.3 ± 1.5 pg/mL 24 h postoperatively, and to 13.2 ± 1.0 pg/mL by day 7. The endotoxin concentrations were higher in recipients who developed complications. Endotoxin is elevated throughout the recipient transplantation procedure and up to 7 days postoperatively. High levels of endotoxin at induction, the anhepatic phase and at certain time points correlated with patients who developed postoperative complications.

## Fifty years ago

**Surbiah N, Stephens FD. Stenotic ureterocele. *ANZ J. Surg*. 1972; 41: 257–63.**

Ureterocle is an intravesical protrusion of the dilated submucosal ureter. It is of infrequent occurrence in children. Ureteroceles arc divided into six types (Stephens, 1971), of which the stenotic variety is the most common. The stenotic ureterocele is confined to the bladder and its orifice is stenotic. Twenty‐six stenotic ureteroceles occurred in eight male and 13 female children. The stenotic ureterocele, localized totally within the bladder, comprised 42% of all types of the 61 in the total series. Fifteen of the ureteroceles occurred in single ureters in 10 patients, five being bilateral, and 11 ureteroceles were located on the ectopic ureter of a double ureter system. Infection caused the presenting symptoms in 11 of 19 patients. The radiographic features were most commonly a basal dome‐like or rounded filling defect with or without the ‘halo’ in the bladder, and on voiding the ureterocele prolapsed into the urethra in two patients. Reflux did not occur into the ureterocele. Histological examination revealed a graduation from full muscle coat with or without hypertrophy to gross deficiency of muscle. The shape of the ureterocele was considered to be developmental and only in part obstructive in origin, accounting for the bizarre siting of the orifice with respect to the terminal end of the ureter and the polypoid configuration. Enlargement of the orifice was effective in seven out of nine ureteroceles, mild reflux occurring postoperatively in two. Transurethral enlargement of the orifice is indicated if the orifice and the presence of peristalsis in the ureterocele have been identified at cystoscopy.

**Hughes ESR, Bennett RC. Caecal pull‐through operations for distal ulcerative colitis: a preliminary report. *ANZ J. Surg*. 1972; 42: 26–30.**

Six patients with distal ulcerative colitis and a relatively normal proximal colon have been treated by colectomy followed by a caecal pull‐through type of anastomosis and preservation of the anal sphincters. The early results in five patients have been quite encouraging and our experience suggests that patients treated by a caecal pull‐through resection have as much chance of achieving a satisfactory postoperative result as many patients treated by ileo‐rectal anastomosis. Though in the early postoperative period anal function may be somewhat deficient and the frequency of bowel actions excessive, both these problems tend to diminish with the passage of time. Furthermore, examination of the retained caecal mucosa suggests that the subsequent re‐exacerbation of symptoms and the development of local or distant complications is less likely to occur in this group of patients. It may be concluded that the procedure is at least a feasible proposition, and the long‐term results of this small group of patients can be awaited with interest. Caecal pull‐through resection for selected patients with severe distal ulcerative colitis may ultimately prove an acceptable alternative to colectomy and ileo‐rectal anastomosis, or even total colectomy and ileostomy, in the management of this condition.

## Seventy‐five years ago

**Hayward JI. The changing outlook in surgery as a result of chemotherapy. II: Thoracic surgery. *ANZ J. Surg*. 1947; 16: 271–6.**

Chest surgery is a new, rapidly developing speciality with a constantly changing outlook, and it would be impossible to say how much of the change has been brought about by advances in surgical technique, anaesthesia and so on, and how much by the advances in chemotherapy. However, it is significant that the decade of chemotherapy for pyogenic organisms which began with sulphanilamide a little more than 10 years ago, coincides with the period of most rapid progress in chest surgery, and there is no doubt that chemotherapy has played an important part. The chemotherapeutic substances which have been employed in chest surgery are mainly the sulphonamides and, more recently, penicillin. A vast number of other substances have been tried, especially in empyema cavities, ranging from bile salts and Metaphen to promin and azochloramide, but they have all been found either unsatisfactory or only of limited value. Streptomycin is eagerly awaited in the hope that it may improve the results and widen the scope of the surgery of pulmonary tuberculosis. None has yet been available in this country, and its discoverers are wisely guarded in their reports because it is too early for them fully to assess its value, so it will not be mentioned further here. Chemotherapeutic substances may be used prophylactically and curatively, and may be administered generally and locally. They have enormous prophylactic value. They are capable of preventing infection in the chest after operations and injuries to a remarkable degree. ‘They have therefore widened the scope and improved the results of major thoracic surgery, especially lobectomy and pneumonectomy. Though very helpful when combined with surgery, chemotherapeutic substances are inefficient at curing infections in the chest which otherwise would be treated surgically.’ To expect them to replace surgical treatment is a false change in outlook. Chemotherapy has changed the clinical picture and course of infective conditions in the chest which respond to it, and our outlook in regard to these conditions has had to change accordingly. A striking example is the ‘cold’ empyema which may follow pneumonia treated with penicillin.

